# Antifungal, Antitumoral and Antioxidant Potential of the Danube Delta *Nymphaea alba* Extracts

**DOI:** 10.3390/antibiotics9010007

**Published:** 2019-12-21

**Authors:** Mihaela Cudalbeanu, Bianca Furdui, Geta Cârâc, Vasilica Barbu, Alina Viorica Iancu, Fernanda Marques, Jorge Humberto Leitão, Sílvia Andreia Sousa, Rodica Mihaela Dinica

**Affiliations:** 1 Faculty of Sciences and Environment, Department of Chemistry Physical and Environment, “Dunărea de Jos” University of Galati, 111 Domnească Street, 800201 Galati, Romania; mihaela.cudalbeanu@ugal.ro (M.C.); geta.carac@ugal.ro (G.C.); 2 Faculty of Food Science and Engineering, Department of Food Science, Food Engineering, Biotechnology and Aquaculture, ‘‘Dunărea de Jos” University of Galati, 111 Domnească Street, 800201 Galati, Romania; vasilica.barbu@ugal.ro; 3 Faculty of Medicine and Pharmacy, Department of Morphological and Functional Sciences, ‘‘Dunărea de Jos” University of Galati, 800008 Romania, 47 Domnească Street, 8000008 Galati, Romania; iancualina.2003@yahoo.com; 4 Departamento de Engenharia e Ciências Nucleares, Instituto Superior Técnico, University of Lisbon, 2695-066 Bobadela, Portugal; fmarujo@ctn.tecnico.ulisboa.pt; 5 IBB-Institute of Bioengineering and Biosciences, Department of Bioengineering, Instituto Superior Técnico, University of Lisbon, 1049-001 Lisbon, Portugal; jorgeleitao@tecnico.ulisboa.pt (J.H.L.); sousasilvia@tecnico.ulisboa.pt (S.A.S.)

**Keywords:** *Nymphaea alba*, antifungal activity, antitumor activity, antioxidant compounds, quercetin

## Abstract

This study aimed to explore for the first time the biological properties such as antifungal, antitumoral and antioxidant of Danube Delta *Nymphaea alba* (*N. alba*) leaf and root methanolic extracts. The toxicity studies of *N. alba* extracts showed no inhibitory effect on wheat seed germination by evaluating the most sensitive physiological parameters (Germination %, Germination index, Vigor index) and using confocal laser scanning microscopy images. The analyzed extracts were found to have high antifungal activity against *Candida glabrata* with MIC values of 1.717 µg/mL for leaf and 1.935 µg/mL for root. The antitumor activity of the both extracts against A2780/A2780cisR ovarian, LNCaP prostate and MCF-7 breast cancer cells was promising with IC_50_ values ranging from 23–274 µg/mL for leaf and 18–152 µg/mL for root, and the combination of *N. alba* extracts with cisplatin showed a synergistic effect (coefficient of drug interaction <1). The antioxidant properties were assessed by β-carotene bleaching, ABTS and FRAP assays and cyclic voltammetry. Quercetin, the most prominent antioxidant, was quantified in very good yields by spectroelectrochemical assay.

## 1. Introduction

Plants are known to be rich sources of bioactive compounds [1]. Generally, plant bioactive compounds differ greatly in terms of their quality and quantity, depending on the plant or on the various constituent parts of the plant [2] and they have been widely assessed for their biological properties [3]. The bioactivity of plant extracts generally depends on the presence of polyphenols and flavonoids, carotenoids, terpenoids or chlorophyll [4]. Thus, new compounds from the plant world can play an important role in the development of more effective and safer drugs to combat infection diseases or cancer [5].

Fungal infections are another major cause of morbidity/mortality worldwide, with invasive fungal disease by members of the *Candida* genus the most common fungal infections in developed countries [6]. Presently available antifungal drug classes are few and some with high toxicity [7]. Furthermore, emerging resistance to the available antifungal drugs has been reported, in particular to azole and echinocandins, due to clinical and environment exposure [8]. Therefore, novel antifungal drugs or adjuvants that lower the quantities of antifungals to achieve eradication of infection with lower toxicity are urgently required [9].

Most active natural biological products are secondary metabolites with complex structures [10], and among them, compounds such as flavonoids, isoprenoids and alkaloids extracted from plants have been exploited for various illnesses due to their pharmacological properties [11], accounting for more than 30% of total antineoplastic drugs [12]. Polyphenols as gallic acid, ellagic acid, and flavonoids such as quercetin and apigenin contribute to antioxidant activity [13,14], and they have gained importance as cytotoxic agents, also promoting apoptosis in cancer cells [15,16].

Cancer treatment modalities involve surgery, chemotherapy and/or radiation therapy [11,17]. Most synthetic chemotherapeutic drugs with antitumor activity present high toxicity, usually associated with undesirable side-effects as is the case of cisplatin, a well-established anticancer drug with non-specific targeting that results in adverse effects and toxicity [18]. The demand for novel antitumor drugs presenting fewer side-effects and better therapeutic outcomes is a priority goal for cancer therapy [19]. Several plant-isolated antioxidant compounds have played an important role in cancer therapy and its prevention [1,13,20]. Although some treatment modalities have succeeded for many patients, cancer remains a major cause of death worldwide [11,21].

**Figure 1:**
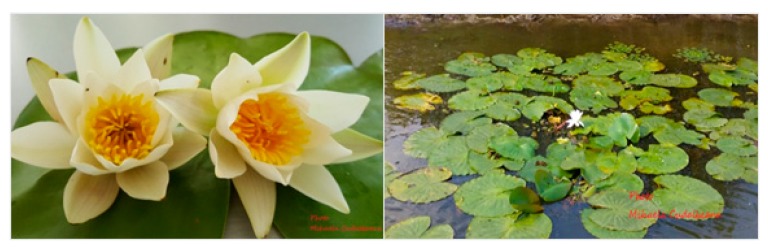
*Nymphaea alba*.

The literature shows that the potential synergy of natural compounds with therapeutic drugs may constitute an important strategy to fight against infections and tumors [2]. With all this in mind, we searched for antifungal, antitumoral, and antioxidant properties in extracts of *Nymphaea alba* (*N. alba*) (Figure 1), a member of a large *Nymphaeaceae* family of aquatic plants worldwide distributed and used for hundreds of years in traditional medicine. *N. alba* species contain antioxidant polyphenols, flavonoids and derived acids such as caffeic acid, ferulic acid, quinic acid, p-coumaric acid, gallic acid, quercetin, luteolin, orientin, catechin, epicatechin, naringin, naringenin and rutin phytochemicals that have shown inhibitory effects on different phases of cell cycle and are also responsible for inducing apoptosis by regulating the expression of different apoptotic signaling pathways [22,23,24]. Flower extracts of the *Nymphaea* genus have been reported to have antineoplastic effects in different types of cancer, including leukemia, lung cancer, colon cancer and prostate cancer [25]. However, there is little evidence of their antineoplastic effects on ovarian and breast cancer cells.

Many phytochemical compounds and various secondary metabolites, such as amino acids, sterols, alkaloids, saponins, tannins, and flavonoids, have been isolated from the *Nymphaea* genus [26,27]. The literature on *Nymphaea* genus has focused on acute and chronic toxicity of phytochemical compounds from crude extracts and their antibacterial, antioxidant, antihelmintic and antidiabetic potential [27]. There are data reports about the medicinal properties of the *Nymphaea* genus, namely the antibacterial potential against human pathogens and plant bacteria, but there is still little scientific evidence for the use of this plant commercially or in a more effective way [26].

The biological evaluation of *Nymphaea alba* has been centered mainly on its antioxidant properties and modulation of the oxidative stress due to its high phenolic content. Many studies have shown that extracts, rich in antioxidants such as polyphenols, have also antimicrobial activities against various pathogens [28] and also against a multitude of opportunistic invaders, including fungi of the *Candida* species [28,29]. In addition, these antioxidants can help prevent disease by reducing oxidative stress in the human body. Regarding the anticancer properties, phenolic compounds can induce cancer cell death and suppress cancer cell progression in a dose-dependent manner. In fact, at low doses these compounds could suppress cellular oxidative stress and cancer cell migration but at higher doses could act as pro-oxidants and anticancer cell proliferation agents [30,31,32].

Herein, we report some relevant biological properties of *N. alba* extracts species from Danube Delta Biosphere Reserve of Romania. The study namely comprises the antioxidant activities of *N. alba* extracts assessed by β-carotene bleaching, ABTS assays, FRAP assays and cyclic voltammetry, the toxicity activity towards wheat seed germination, the antifungal properties against *Candida glabrata* strain, and the cytotoxic activity on ovarian, breast and prostate cancer cells.

## 2. Results

### 2.1. Toxicity Effects on Wheat Seed Germination

Wheat (*Triticum aestivum* L.) seed germination has been used as a very sensitive model to measure the toxicity of different compounds [33]. Considering the literature data, we investigated the toxicity of the *N. alba* leaf and root extracts on wheat seed germination in order to evaluate the hazardous potential of these extracts in case of potential uses in the pharmaceutical industry. The most sensitive physiological parameters evaluated were selected based on the literature data. Higher plants are suitable for toxicity studies, as they are recognized as excellent indicators of cytogenetic and mutagenic effects of chemicals. The germination tests are very simple, not very time consuming, and cheap, and could therefore be an ideal method for testing the toxicity of chemicals [34,35].

The results revealed that the application of the different concentrations of *N. alba* extracts had no toxic influence on seed germination. Compared to the control, the shoot and root of the wheat germinated seeds with different concentrations of *N. alba* extracts showed a slightly greater size, which may be due to the stimulant action of the bioactive compounds (Table 1).

**Figure 2:**
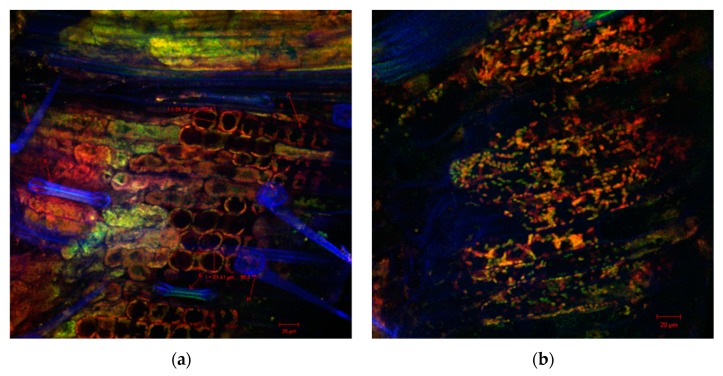
Confocal laser scanning microscopy images of the wheat sprout sections variants: (**a**) ultrapure water control samples; and (**b**) methanol control samples.

**Figure 3:**
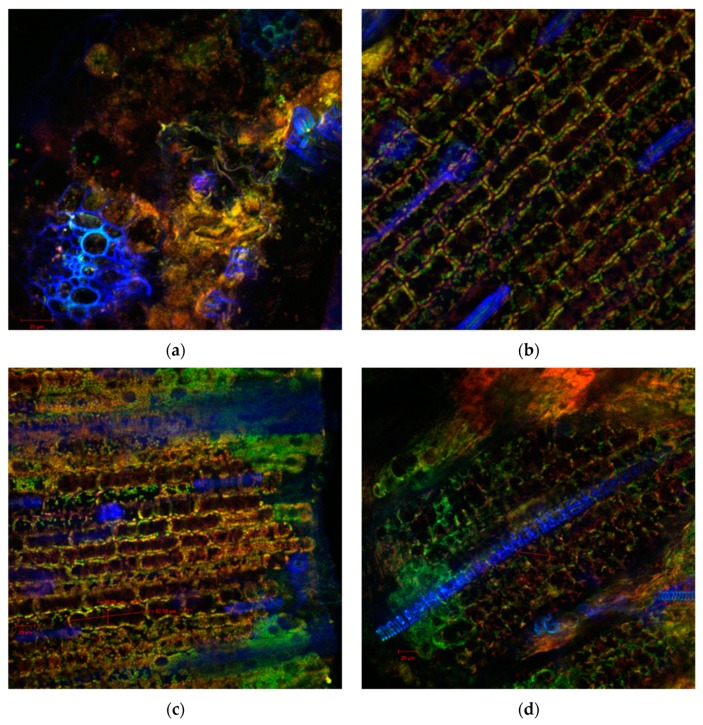
Confocal laser scanning microscopy images of the wheat shoot sections variants of the *N. alba* leaf extract treatments: (**a**) 10 µg/mL, (**b**) 100 µg/mL, (**c**) 500 µg/mL, and (**d**) 1000 µg/mL.

**Figure 4:**
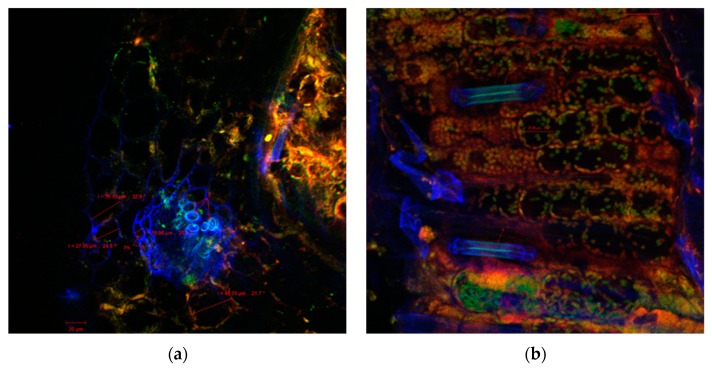

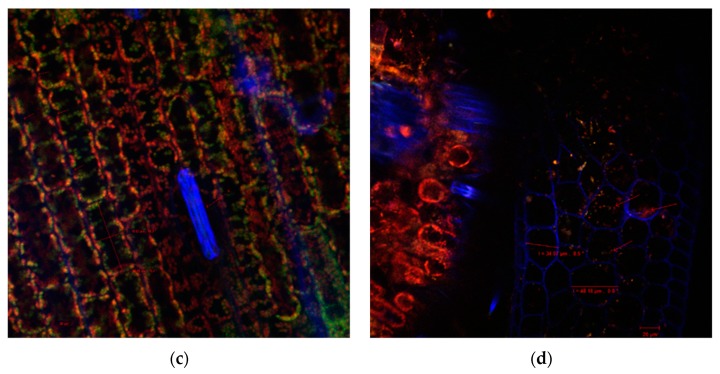
Confocal laser scanning microscopy images of the wheat shoot sections variants of the *N. alba* root extract treatments: (**a**) 10 µg/mL, (**b**) 100 µg/mL, (**c**) 500 µg/mL, and (**d**) 1000 µg/mL.

Also, the sections through the stems of the seedlings of the wheat germinated seeds with both extracts indicated healthy plant tissues, without morphological and structural alterations, as was observed by confocal laser scanning microscopy (Figure 2, Figure 3 and Figure 4). Cellular differentiation was not influenced by *N. alba* leaf and root extracts used in the experiments. From microscopic analysis in all samples, normal structures can be observed, including: the monolayered epidermis, the cortical parenchyma with isodiametric cells, the assimilator tissue rich in chloroplasts, the pericycle separating the cortical parenchyma from the central stele (Figure 4d), and the specific vascular tissue components (in blue): xylem (X) (Figure 2a, Figure 3a,d and Figure 4a), or phloem (Ph) (Figure 4a). The confocal analysis from the experimental variants showed normal differentiated epidermal cell types such as numerous trichomes or hairs (H) or stomata type Zea (S) (in blue), indicating that the *N. alba* leaf and root extracts enabled or even facilitated the differentiation of these structures. The only changes reported at higher concentrations of *N. alba* leaf and root extracts were the tendency to elongate the cells, which become easy prosenchymatous, with a long side of 46.85 μm (Figure 3c) or of 82.5 μm (Figure 4c). The presence of a greater number of plasmodesmata (see the arrows in Figure 3b and Figure 4c) allow a better intercellular communication, probably due to interaction of plant tissue with phytochemical compounds from *N. alba* extracts. When a concentration of 1000 µg/mL of the *N. alba* root extract (Figure 4d) was used, corpuscles (see the red arrows) with autofluorescence in the range 570–650 nm were identified in the cytoplasm of parenchyma cells, which might result from the metabolism/assimilation of biologically active compounds from extracts used for seed germination.

### 2.2. Antifungal Activity

**Figure 5:**
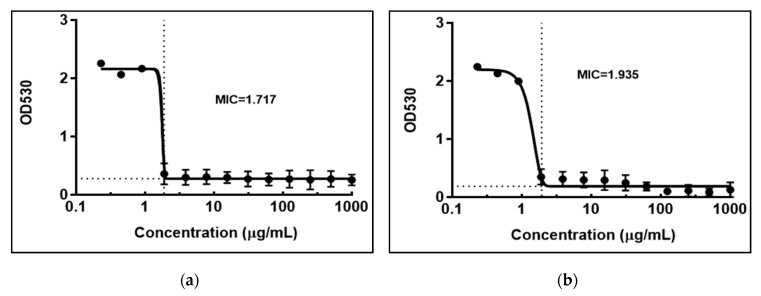

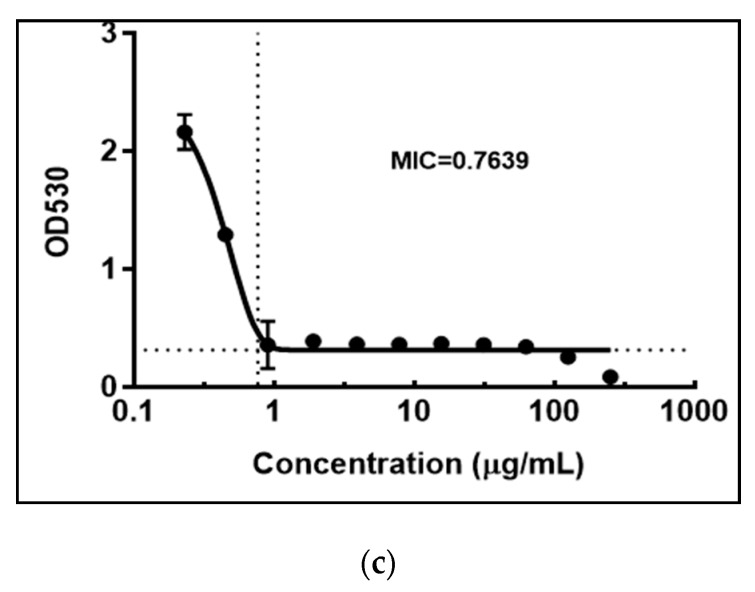
The MIC of the *N. alba* leaf (**a**) and root (**b**) extracts and fluconazole (**c**) against *C. glabrata* CBS 138.

Compounds that have both anticancer and antimicrobial activity are promising therapeutic agents due to their potential to diminish the occurrence of opportunistic fungal infections often associated with chemotherapy [36]. The antifungal activity (MIC) was evaluated in vitro against 4 fungal strains *C. glabrata* CBS 138, *C. albicans* SC 5134, *C. parapsilosis* ATCC22019 and *C. tropicalis* ATCC750 using *N. alba* extracts with concentrations between 0.23 and 2000 µg/mL, and fluconazole (FLC) as control with concentrations between 0.23 and 250 µg/mL (Figure 5). The *N. alba* leaf and root extracts were found to be active against *C. glabrata*, with MIC values of 1.717 and 1.935 μg/mL for leaf and root extracts, and 0.7639 for FLC, respectively [37]. The obtained results showed that the action of the extracts was specific for *C. glabrata,* having no action on the other *Candida* strains tested.

*C. glabrata* is phylogenetically, genetically and phenotypically distinct from the other pathogenic yeast species like *C. albicans*, *C. parapsilosis* and *C. tropicalis* [38]. The MIC values of the *N. alba* leaf and root extracts against *C. albicans* SC 5134, *C. parapsilosis* ATCC22019 and *C. tropicalis* ATCC750 were higher than 2000 µg/mL.

**Figure 6:**
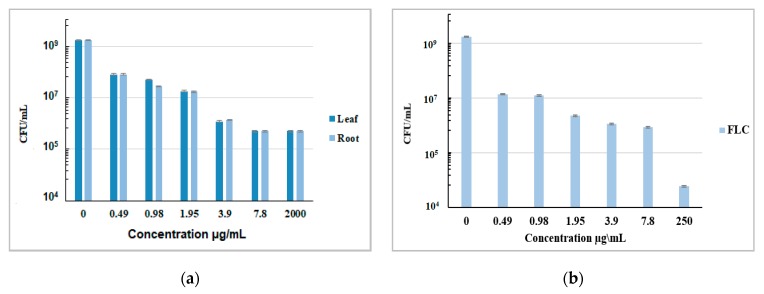
CFUs of *C. glabrata* CBS 138 strain, cultured in the presence of the indicated concentrations of the *N. alba* leaf and root (**a**) extracts and fluconazole (**b**).

The antifungal effects of the *N. alba* leaf and root extracts and FLC against *C. glabrata* CBS 138 strain were confirmed by viable count (CFU/mL). The results presented in Figure 6 show that both extracts exhibit fungistatic activity against *C. glabrata* CBS 138.

The probable mechanism of action implicated in this type of compound, present in *N. alba* extracts, is the inhibition of efflux pumps, inhibition of fungal growth, reduction in number of yeast cells and germ tubes which ultimately results in induction of cell death or apoptosis [39].

### 2.3. Cytotoxic Effects of N. alba Extracts on Normal and Tumor Cells

Natural bioactive products can be considered prospective chemotherapeutic drugs due to their expected less toxic profile [15].

Compounds of plant origin have been shown to be effective against LNCaP prostate tumor cell growth [40]. However, the active species were identified mainly as fatty acid derivatives. A mixture of phytochemical compounds with a multitude of biological activities could have additional or synergistic effects against breast cancer [41]. In this regard, studies by Ashidi et al. [42] reported that the methanolic extracts of African medicinal plants have significant cytotoxicity on MCF7 breast cells, but also present some toxicity towards normal cells. Previous studies on phenolic constituents of *Nymphaea alba* extracts reported anti-proliferative activities and the ability to suppress cancer cell invasion but in different tumor models other than the MCF7 cells [31,32]. Aimvijarn et al. [32] reported that *Nymphaea* extract showed cytotoxicity to B16 melanoma cells with IC_50_ = 814 µg/mL. The extract at 800 and 1000 µg/mL demonstrated pro-oxidant activity related to cell apoptosis.

**Figure 7:**
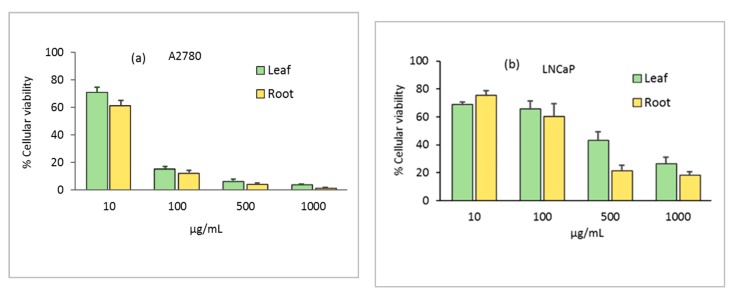

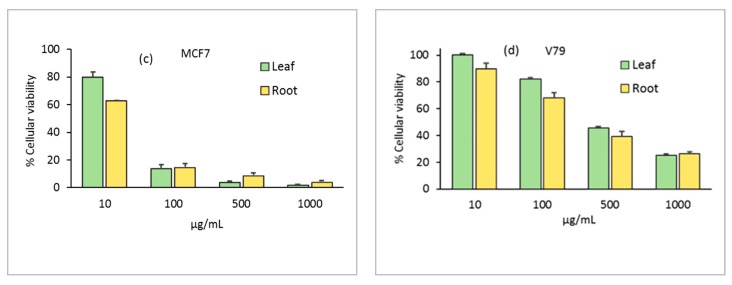
In vitro cytotoxicity assessment of *N. alba* leaf and root extracts on A2780 (**a**), LNCaP (**b**), MCF7 (**c**), and V79 (**d**) cell lines.

In our search for therapeutic agents based on plants, we evaluated the effect of the crude extracts from leaf and root of the *N. alba* species against tumor cell lines and on a normal cell line, using the MTT assay. The cytotoxicity of *N. alba* leaf and root extracts on the cells was assessed at various concentrations in the range 10–1000 µg/mL. Upon treatment with both extracts at concentrations higher than 100 μg/mL for 24 h, the cellular viability of the A2780 ovarian cancer cells was lower than 20% (Figure 7a). In the LNCaP prostate cells, the cytotoxicity of *N. alba* extracts was lower than that observed for the ovarian cells. However, the effect was dose dependent, and for concentrations higher than 500 μg/mL, the root extract exhibited percentages of cellular viability of ca. 13–20% (Figure 7b). The cytotoxicity of *N. alba* leaf and root extracts towards the MCF7 cells was also analyzed. The results showed that *N. alba* extracts significantly inhibit the growth of MCF7 cancer cells at concentrations higher than 100 μg/mL, in a similar mode to that observed for the ovarian cells (Figure 7c).

Our results indicated that in all cancer cell lines studied, the cytotoxicity was high, with IC_50_ values in the range of 23–274 µg/mL for the leaf and 18–152 µg/mL for the root (Table 2). Moreover, the root extracts had a slight tendency to be more cytotoxic, as can be observed in Figure 7.

Results presented herein encourage us to continue our research on the assumption that *N. alba* extracts contain promising therapeutic agents. Leaf and root extracts were also tested in the normal V79 cells at 10–1000 μg/mL to assess their cytotoxicity (Figure 7d). In these cells, a significant loss of cellular viability (<50%) was observed only at concentrations higher than 500 μg/mL, which indicate that the extracts have an important selectivity for cancer cells.

The cytotoxicity of the *N. alba* leaf and root extracts, due to the presence of bioactive compounds as corilagin, chlorogenic acid or caffeic acid can be explained on the one hand by inhibition of gene β-catenin and induction of genes as GSK-3β, promoting the tumor cell apoptosis [23,24]. Flavonoids such as quercetin, naringin, naringenin, and apigenin inhibit or activate the different phases of cell cycle, and are also responsible for inducing apoptosis by regulating the expression of different apoptotic signaling pathways. Most of them show inhibitory effects on breast cancer by down-regulating estrogen receptor ER-α expression, inhibiting metastasis and proliferation, inducing caspase-mediated cell death and arresting the cell cycle [23,24,43].

The IC_50_ value is a highly sensitive metric that is used to evaluate the results of drug response and drug potency. The IC_50_ values of the *N. alba* extracts against normal and cancer cell lines were calculated and are shown in Table 2. The results demonstrate that the *N. alba* leaf and root extracts have important cytotoxicity against A2780 and MCF7 cells, but exhibit negligible toxicity against the LNCaP and the V79 cell lines. Among the three cancer cell lines, the A2780 and MCF7 cell lines seem to be more sensitive to treatments with *N. alba* extracts than the LNCaP cell line. The two extracts are cell type selective and, most importantly, are less cytotoxic for normal cells. However, it is important to note that chemotherapeutic drugs act against cancer cells in a non-selective way, leading to adverse side effects and drug resistance. Combination therapies have proven to be promising alternatives for cancer therapy. As previously described [44], plant extracts contain several beneficial compounds that can target multiple pathways in cancer cells and can be more selective, sparing the normal cells. In addition, such combinations will enable a reduction in the dose of chemotherapy drugs.

#### Combined Cytotoxic Effects of *N. alba* Extracts and Cisplatin on Ovarian Cell Lines

The disadvantage of many cytotoxic agents is their high toxicity, which can produce considerably toxic side effects [15,45], not only on tumor cells but also on healthy cells [17]. Combined therapies in which both agents exhibit cytotoxicity result in dose-limited effects [46]. Cisplatin is one of the most effective chemotherapeutic agents used in the treatment of cancer, being a cytotoxic drug that affects the DNA [47]. Tumor resistance to cisplatin-based drugs is an obstacle in the treatment of, for instance, ovarian cancer. Novel approaches for treating ovarian cancer are the use of natural compounds with antineoplastic effects in combination with platinum-based drugs [48]. Two or more drugs that individually produce similar effects are expected to have greater effects when administered in combination. The combined effect can typically be described as synergistic, additive, indifferent or antagonistic. When the combined effect is greater than the individual effect of each drug, the combination is considered synergistic [49]. To reduce the toxic effects of drugs on normal tissues, combinations of lower doses of individual chemotherapeutic agents are required [50].

To evaluate the potential of the *N. alba* extracts by means of the above-mentioned combined therapy, we used the most effective extracts for the A2780 sensitive tumor cell line and extracts that do not show high cytotoxicity on normal cells. The *N. alba* leaf and root extracts were combined with cisplatin and were incubated with the A2780 and A2780cisR (cisplatin-resistant) cancer cell lines for 24 h. Experiments were also performed using either cisplatin or *N. alba* extracts. To assess the in vitro cytotoxicity of *N. alba* extracts combined with cisplatin on the A2780 and A2780cisR cell line, a ratio of 1:10, cisplatin: extract was used. The extracts were used in concentrations of 10, 100, 500 and 1000 μg/mL and the cisplatin was used in concentrations of 1, 10, 20, 50, and 100 μg/mL.

**Figure 8:**
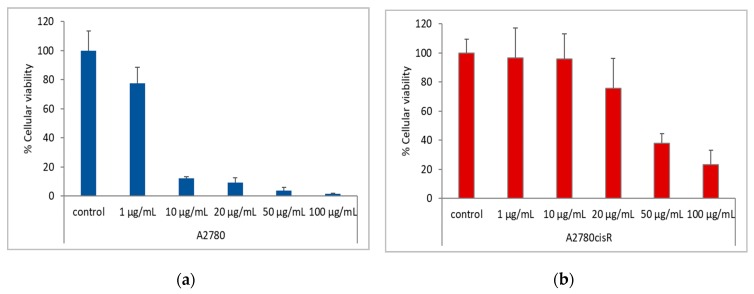
In vitro cytotoxicity assessment of cisplatin on A2780 (**a**) and A2780 cisR (**b**) cell lines. The IC_50_ values found using the GraphPad Prism software (ver. 5) were: A2780, 2.4 ± 0.6 µg/mL (7.9 µM) and A2780cisR, 27 ± 13 µg/mL (89 µM). Control = cells without treatment. Data are mean ± SD of two independent experiments done with at least four replicates per condition.

The experiments presented herein showed that adding *N. alba* extracts containing antioxidants permit reducing the concentration of cisplatin in order to decrease A2780/A2780ciR cell survival (Figure 8).

The synergistic interactions between *N. alba* extracts and cisplatin were performed using the Chou method [51]. The combination index (CI) is given in Table 3. The obtained results indicate CI values ranging from 0.05 to 0.5, suggesting a strong synergism in the growth inhibitory effect.

### 2.4. Antioxidant Capacities by Spectrophotometric Methods

Different assays were used to determine the antioxidant activities of the *N. alba* leaf and root extracts, since antioxidant activities of different types of compounds involve different mechanisms, and a single assay is not conclusive. Thus, the antioxidant capacities of the *N. alba* leaf and root extracts were measured three times to test the evaluated reproducibility by a series of a microplate reader assays: β-carotene bleaching (BCB), ABTS, and FRAP. A lower IC_50_ value indicates increased antioxidant activity. Concentrations of the *N. alba* leaf and root extracts between 1–500 μg/mL were used for the antioxidant assays and their IC_50_ values. The obtained results for BCB, ABTS and FRAP assays are shown in Table 4. All data were determined for the sample at a concentration of 500 μg/mL.

The β-carotene bleaching assay is used to measure the antioxidant activity for the plant extracts, especially to investigate lipophilic antioxidants. This assay measures the inhibition of autooxidation of the linoleic acid and β-carotene [52]. β-carotene forms a complex with linoleic acid and oxidizes it. The consumption of β-carotene causes the reduction of the bright yellow color of β-carotene. The presence of an antioxidant in the reaction mixture inhibits the consumption of β-carotene through inactivation of the linoleic acid free radicals, thus leading to the release of β-carotene from the complex with linoleic acid and the recovery of the yellow color of the reaction mixture. Results presented in Table 1 show a 78.6% inhibition of β-carotene consumption for the leaf extract, and 90.1% for the root extract. The antioxidant activity presented IC_50_ values as low as 33 μg/mL for the leaf extract and 21 μg/mL for the root extract.

The ABTS assay is a useful tool for the determination of the antioxidant activity of lipophilic and hydrophilic compounds. The ABTS radical cation reacts rapidly with antioxidant compounds because of its solubility in both aqueous and organic solvents [53]. The *N. alba* extracts possessed a high antioxidant capacity for ABTS inhibition, with IC_50_ values as low as 10 μg/mL for the root extract and 12 μg/mL for the leaf extract. The percentage of ABTS inhibition was 78.2% for the root extract and 77% for the leaf extract.

The FRAP assay relies on the ability of antioxidants to reduce iron (III) to iron (II), through the reaction with 2,4,6-tris(2-pyridyl)-s-triazine, forming a violet blue color [54]. The FRAP assay provides a direct estimation of the reductants in a sample and it is based on the ability of the analyte to reduce the Fe^3+^/Fe^2+^ couple. The *N. alba* extracts showed good ferric reducing antioxidant power, being the scavenging activity of the leaf and root extracts 74.6% and 79.7%, respectively. The activity presented IC_50_ values as low as 30 μg/mL for the leaf extract and 13 μg/mL for the root extract.

The root extract exhibited a higher antioxidant activity (and a lower IC_50_ values) than the leaf extract. Consistently, Trolox equivalent values for the BCB, ABTS and FRAP assays were higher for the root extract than for the leaf extract. Trolox equivalent values for the root extract were as follows: 79,371 µg TEq/1 g (BCB assay), 1950 µg TEq/1 g (ABTS assay) and 2407 µg TEq/1 g (FRAP assay). Trolox equivalent values for the leaf extract were as follows: 28,933 µg TEq/1 g (BCB assay), 1309 µg TEq/1 g (ABTS assay) and 2245 µg TEq/1 g (FRAP assay). Our results are in accord with those of the literature data, which showed strong scavenging activity of DPPH radical for the aqueous and ethanolic flower and rhizome extracts of *Nymphaea alba* [55,56,57].

In summary, the BCB, ABTS and FRAP measurements indicate the stability of the bioactive compounds from *N. alba* leaf and root extracts, which means that the antioxidant activities and IC_50_ values remain constant and act for a long period of time.

### 2.5. Electrochemical Evaluation of Antioxidant Capacity

**Figure 9:**
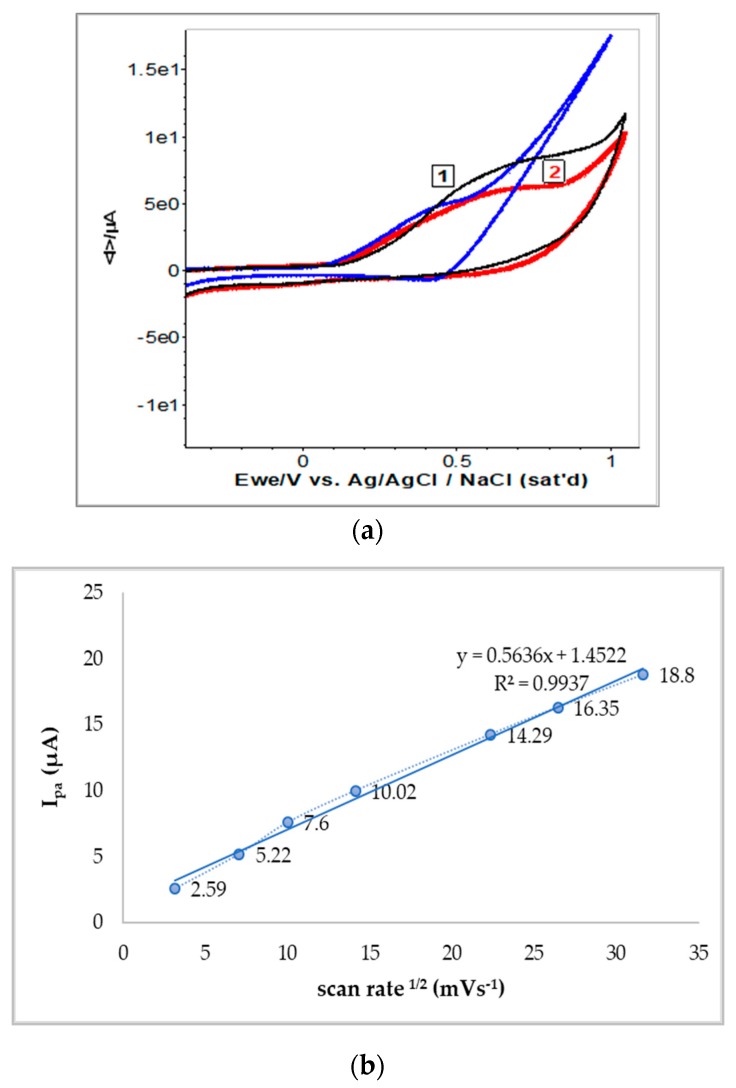
Cyclic voltammograms recorded for the *N. alba* leaf (1) and root (2) extracts compared with quercetin (blue line). E = ± 1 V vs. Ag/AgClsat., scan rate of 100 mV s^−1^ (**a**). Dependence of the anodic current vs square root of the scan rates (**b**).

Electrochemical investigations of both *N. alba* extracts (1 mg/mL in methanol) were performed using cycling voltammetry and UV-Vis spectroscopy. Samples showed light positive potential from the open circuit potential measurements, with the leaf extract presenting a potential about 30 mV more positive when compared to the root extract. The cyclic voltammograms of the root extract revealed moderate electrochemical activity compared with those obtained from the leaf extract. The *N. alba* extracts exhibited an intensity of anodic current (Ipa) up to 10.20 μA for the leaf extract and up to 6.32 μA for the root extract. The quercetin (10^−3^ M), as the major antioxidant compound, showed similar voltammogram profile as in the *N. alba* extracts (Figure 9a, blue line). The E_1/2_ of leaf and root extracts was 0.410 V ± 10 mV compared with the quercetin potential [58].

**Figure 10:**
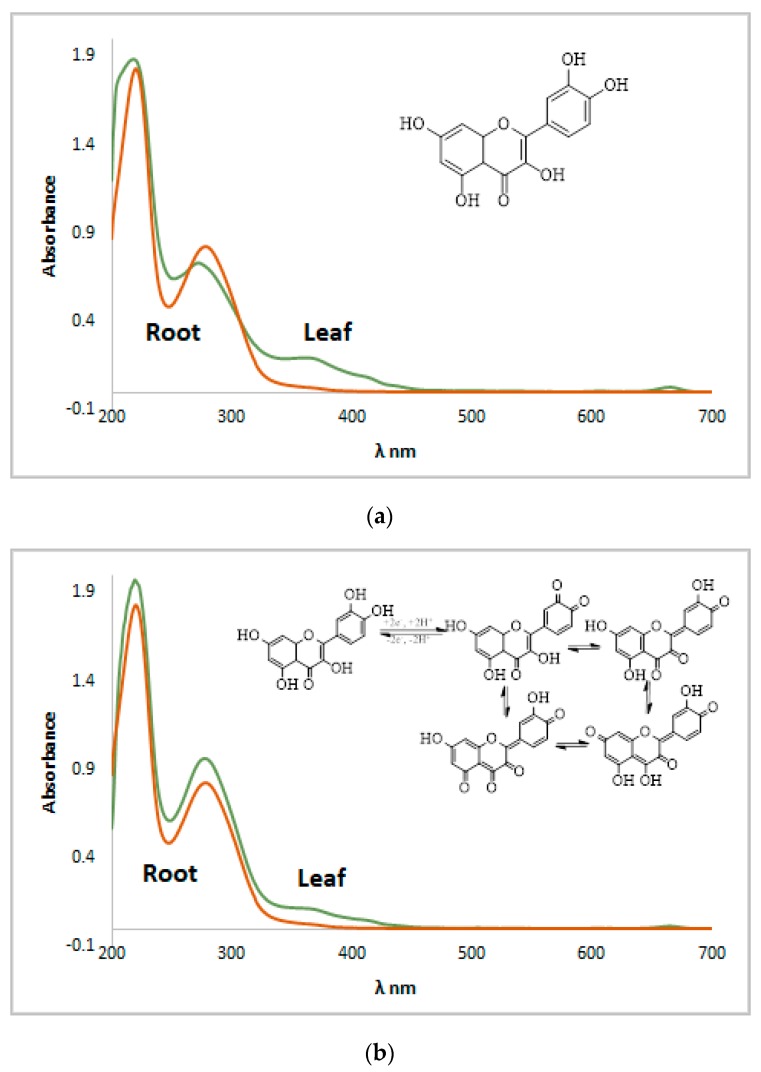
UV-VIS spectra recorded for the *N. alba* leaf and root methanolic extracts before (**a**) and after cyclic voltammetry experiments (**b**).

Five consecutive voltammograms were recorded at different sweep rates (10–1000 mVs^−1^) and the current intensity increased with scan rate. No asymmetry was observed between currents (I_pa_/I_pc_), an indicative of quasireversible process for the antioxidant compounds. The intensity of the anodic current varies linear with the scan rate due to the typical diffusion control processes (Figure 9b). Subsequently, UV-Vis spectra of both extracts were recorded before and after electrochemical measurements (Figure 10). Spectra profiles were almost similar for both *N. alba* extracts (Figure 10a). These results confirm the existence of antioxidant compounds in the extracts, as flavonoids range between 240–400 nm. The *λ*_max_ of quercetin is at 385 nm [58], and its oxidative degradation exposed to electrochemical measurements is highlighted in Figure 10b.

Therefore, the observed electrochemical process supports the relevant antioxidant activity of *N. alba* extracts, which can be attributed to quercetin [22,58], one of the flavonoids present in the leaf extracts.

**Figure 11:**
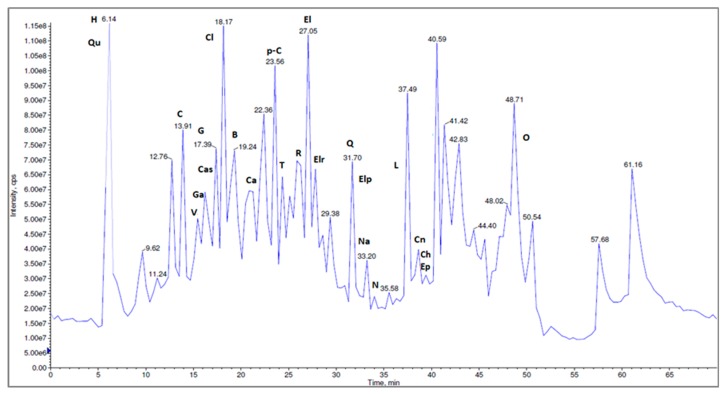
The LC-MS/MS chromatographic separation of the methanolic *N. alba* leaf extract (H-HHDP-hexoside, Qu–quinic acid, C–corilagin, V–vanillic acid, Cas–castalin, Ga–gallic acid, G–geraniin, Ca–caffeic acid, p-C–coumaric acid, T–tannic acid, R–rutin, El–ellagic acid, Elr–ellagic rhamnosyl acid, Elp–ellagic pentoside acid, Cn–cinnamic acid derivative, Na–naringenin, N–naringin, Ch–catechin, Ep–epicatechin, Cl–chlorogenic acid, Q–quercetin, L–luteolin, B–brevifolin, O–orientin).

The presence of quercetin was previously confirmed by HPLC-MS/MS analysis using quercetin as standard [22]. The identification was performed based on their characteristic [M-H]^−^ and MS/MS fragmentation profile, according to the literature data, the MS spectra providing a parent ion at 301 m/z, and a fragment ion at 150 m/z. Using reference standards, the chromatographic analysis (Figure 11) has led to the identification of different fragments of other polyphenolic compounds as phenolic acids, flavonoids, tannins, and other non-flavonoid polyphenols [22].

Overall, 27 bioactive compounds (caffeic acid, p-coumaric acid, chlorogenic acid, naringin, naringenin, vanillic acid, quercetin, rutin, HHDP-hexoside, corilagin, tannic acid, gallic acid, ferulic acid, ellagic acid, ellagic acid pentoside, quinic acid, castalin, orientin, apigenin, luteolin, brevifolin, cinnamic acid derivative) are reported for the methanolic extracts of *N. alba* from the Danube Delta landscape; of these, 26 were found in leaf extracts and 22 in root extracts [22].

Compared with the root extract, the leaf extract contains four additional compounds— chlorogenic acid, quercetin, luteolin and cinnamic acid derivative—but the root contains two compounds that are missing from the leaves: apigenin and ferulic acid. All of these bioactive compounds were found to exert a variety of pharmacological effects, including antitumoral, antimicrobial, and antioxidant (Table 5). The mechanism of action implicated in this type of compound is through inhibition of efflux pumps, inhibition of fungal growth, reduction in number of yeast cells and germ tubes, which ultimately results in induction of cell death or apoptosis [39]. Therefore, these structural differences between phytochemicals of two extracts could explain the differences between the biological activities.

## 3. Materials and Methods

### 3.1. General

All the reagents and organic solvents used for analysis were purchased from Sigma Aldrich (Steinheim, Germany). Absorbance and fluorescence measurements for antioxidant activities were done using a multiwell plate reader (Tecan Pro 200, Tecan Trading AG, Männedorf, Switzerland). The *N. alba* plant was collected in June 2017, from the Somava-Parches Lagoon Complex, situated in the Danube Delta Biosphere Reserve of Romania. Specimens were deposited at the Botanical Garden of Galati, Romania [22].

### 3.2. Preparation of N. alba Extracts

Leaf and root of the *N. alba* species were dried at room temperature until completely drying and then finely ground. 10 g of ground powder plant was extracted with 100 mL of methanol for 2 h, by a mass/volume ratio of 1/10 (w/v) using an ultrasonic bath. The extracts were filtered initially through cotton and then through quantitative filter paper and then concentrated under reduced pressure in a rotary evaporator to remove the solvent. The obtained crude extracts were stored refrigerated at 4 °C until further use.

### 3.3. Toxicity Evaluation of N. alba Extracts on Wheat Seed Germination

Prior to germination the wheat seeds were surface-sterilized for about 5 min with 1% sodium hypochlorite solution to prevent fungal growth, followed by washing for five times with ultrapure water. Twenty-five uniform and disinfected wheat seeds were selected and incubated in germination boxes with double layers of paper filter in the presence and in the absence of different concentrations (10, 100, 500 and 1000 µg/mL) of *N. alba* leaf and root extracts. Four replicates of 25 seeds were used for each sample and control. Control seeds were treated with distilled water only. The germination boxes were kept in darkness at room temperature (24 °C) for 5 days. In the germination process, the wheat seeds with a root longer than 1 mm were considered to be germinated. After 5 days, the shoot and root length of wheat seeds were measured (in mm). The germination index (GI) was obtained by dividing the germination percentage (G %) by the relative root growth (RRG %). The vigor index (VI) was also obtained. Parameters were calculated as follows [90]:

(1) $$\mathrm{GI}=\frac{G\%}{\mathrm{RRG}\%}$$

(2) $$G\%=\frac{\mathrm{No}.\;\mathrm{of}\;\mathrm{germinated}\;\mathrm{wheat}\;\mathrm{seeds}\;\mathrm{in}\;\mathrm{sample}}{\mathrm{No}.\;\mathrm{of}\;\mathrm{germinated}\;\mathrm{wheat}\;\mathrm{seeds}\;\mathrm{in}\;\mathrm{control}}\;*100$$

(3) $$\mathrm{RRG}\%=\frac{\mathrm{Mean}\;\mathrm{root}\;\mathrm{length}\;\mathrm{in}\;\mathrm{sample}}{\mathrm{Mean}\;\mathrm{root}\;\mathrm{length}\;\mathrm{in}\;\mathrm{control}}*100$$

(4) $$\mathrm{VI}=G\%*\frac{\mathrm{Mean}\;\mathrm{of}\;\mathrm{root}\;\mathrm{length}}{\mathrm{Mean}\;\mathrm{of}\;\mathrm{shoot}\;\mathrm{length}}$$

Tolerance index (TI) combines several individual measurement parameters and generates an index between 0 and 1. A low TI indicates a high susceptibility to biotic or abiotic stresses originating from the *N. alba* leaf and root extracts. The TI a and TI b were calculated using [91]:

(5) $$\mathrm{TI}\;a=\frac{\mathrm{Germination}\;\mathrm{rate}\;\mathrm{of}\;\mathrm{treated}\;\mathrm{sample}\;\left(\%\right)}{\mathrm{Germination}\;\mathrm{rate}\;\mathrm{of}\;\mathrm{control}\;\left(\%\right)}*\frac{\mathrm{Root}\;\mathrm{length}\;\left(\mathrm{mm}\right)\mathrm{of}\;\mathrm{treated}\;\mathrm{sample}}{\mathrm{Root}\;\mathrm{length}\;\left(\mathrm{mm}\right)\;\mathrm{of}\;\mathrm{control}}$$

(6) $$\mathrm{TI}\;b=\frac{\mathrm{Germination}\;\mathrm{rate}\;\mathrm{of}\;\mathrm{treated}\;\mathrm{sample}\;\left(\%\right)}{\mathrm{Germination}\;\mathrm{rate}\;\mathrm{of}\;\mathrm{control}\;\left(\%\right)}*\frac{\mathrm{Dry}\;\mathrm{matter}\;\left(g\right)\;\mathrm{of}\;\mathrm{treated}\;\mathrm{sample}}{\mathrm{Dry}\;\mathrm{matter}\;\left(g\right)\;\mathrm{of}\;\mathrm{control}}$$

#### Confocal Laser Scanning Microscopy (CLSM)

The germination of the wheat seeds was followed by confocal laser scanning microscopy using a Zeiss system (LSM 710) equipped with a diode laser (405 nm), Ar-laser (458, 488, 514 nm), DPSS laser (diode pumped solid state e 561 nm) and HeNe-laser (633 nm). To estimate the possible toxic effects of the extracts on the wheat germs, wheat seeds were germinated in the presence of different concentrations of the *N. alba* leaf and root extracts. The microscopic glass slides with sections through root and shoot of samples were observed using a Zeiss AxioObserver Z1 inverted microscope equipped with a 40× apochromatic objective (numerical aperture 1.4) and the FS49, FS38 and FS15 filters. The parameters used for image acquisition were line scan mode, mean method, average number 4, speed 6, 12-bit depth. The images were analyzed with the ZEN 2012 SP1 software (Black Edition).

### 3.4. Assessment of Antifungal Activity of N. alba Extracts

#### 3.4.1. Determination of *N. alba* Extracts’ Minimum Inhibitory Concentration (MIC)

The antifungal activity of the *N. alba* extracts against *Candida glabrata* CBS 138, *Candida albicans* SC 5134, *Candida parapsilosis* ATCC22019, and *Candida tropicalis* ATCC750 was assessed based on the determination of MIC values, defined as the lowest concentration that inhibited fungal growth. The fluconazole (FLC) was used as control. MIC values were experimentally determined in a 96 well plate using RPMI 1640 medium (Sigma) containing 20 g/L glucose (final concentration), and buffered to pH 7.0 with 0.165 M morpholine propane sulphonic acid (MOPS; SIGMA) using the broth microdilution method recommended by EUCAST (European Committee on Antimicrobial Susceptibility Testing) for Candida spp. [92], and as previously described [9,93]. The extracts were sequentially 1:2 diluted in order to obtain final concentrations ranging between 0.23 and 2000 µg/mL. The concentration of the FLC ranged between 0.23 and 250 µg/mL After inoculation, the 96-well round-bottom polystyrene microtiter plates (Greiner Bio-One) were incubated at 35 °C for 24 h and the optical density was determined at 530 nm using a SpectrostarNano microplate reader (BMG Labtech, Freiburg, Germany). The experiments were done in triplicate, and MICs were calculated as the mean values.

#### 3.4.2. Assessment of Fungicidal/Fungistatic Activity

To assess the fungistatic or fungicidal activity of the extracts against *C. glabrata* CBS 138, the CFUs were counted for the wells containing the concentrations of extracts above and below the estimated MIC. For this purpose, 100 µL of each cell suspension of *C. glabrata* CBS 138 treated with *N. alba* leaf and root extracts were serial diluted (1:10) in NaCl 0.9% and were plated in Yeast Extract-Peptone-Dextrose (YPD) Agar plates. The plates were incubated for 24 h at 30 °C and the total number of CFUs was determined. The results are presented as Log10 CFU per mL (Log10 CFUs/mL).

### 3.5. In Vitro Cytotoxicity Assessment of N. alba Extracts on Normal and Tumor Cell Lines

The effect of *N. alba* extracts on the cellular viability of A2780/A2780cisR ovarian (sensitive and resistant to cisplatin) (Sigma-Aldrich, Darmstadt, Germany), MCF7 breast (hormone dependent) (ATCC), LNCaP prostate (hormone dependent) (ATCC) cancer cells, and V79 normal lung fibroblasts (ATCC), were measured using the MTT assay (MTT = 3- (4,5-dimethylthiazol-2-yl) -2,5-diphenyltetrazolium bromide reagent) based on the conversion of the tetrazolium salt to formazan by metabolic active cells. Cells were grown in DMEM + Glutamax I (V79 and MCF7) or RPMI 1640 (LNCaP, A2780/A2780cisR) media, supplemented with 10% fetal bovine serum (FBS) and 1% penicillin/streptomycin. For the assays, cells were seeded in 96-well plates and left 24 h to adhere in a 5% CO2 incubator at 37 °C. 200 μL of cell culture media with the extracts at serial dilutions were applied to each well. After 24 h of incubation, the supernatant was removed, MTT (0.5 mg/mL PBS) was added, and the plates were incubated for 3 h at 37 °C. The purple formazan formed was dissolved in 200 μL DMSO. Optical density was measured at 570 nm using a microplate reader (Power Wave Xs, Bio-Tek, Winooski, USA).

#### Analysis of Combined *N. alba* Extracts and Cisplatin Cytotoxic Effects

For the analysis of the combined effects of *N. alba* extracts and cisplatin, the concentration ratio of the two drugs was set from the MTT assays on the A2780 and A2780cisR cancer cell lines. Five doses of *N. alba* extract and cisplatin were used from serial dilutions. The combined method was used for the evaluation of the potential synergism between *N. alba* extract and cisplatin [58]. The combination index (CI) was calculated by the CompuSyn software and CI < 1, CI = 1, and CI > 1, indicated synergy, additive effect, and antagonism, respectively [46,94,95].

### 3.6. Antioxidant Capacity Assays

#### 3.6.1. β-Carotene Bleaching

β-Carotene bleaching was assessed by the measurement of the inhibition of formation of volatile organic compounds and the diene conjugated hydro-peroxides, resulting from the oxidation of linoleic acid. A solution of 2 mg of β-carotene in 1 mL of chloroform was introduced into a round bottom flask containing 2 mg of linoleic acid and 200 mg of Tween 40. After the chloroform evaporation, 100 mL of distilled water saturated with oxygen were added with vigorous stirring. 100 µL from this new solution was added in a 96-well plate containing concentrations between 1–500 μg/mL of extract samples. Distilled water was used as the negative control. Absorbance was measured at 460 nm at different intervals of time (30 min, 60 min, 120 min and 180 min). A linear standard curve of Trolox was made, using amounts of Trolox between 0 and 250 µg. Results are expressed as µg TEq/g sample [47,96].

#### 3.6.2. ABTS Assay

The ABTS radical cation stock solution was prepared by mixing 7.8 mM 2,20-azinobis (3-ethylbenothiazoline-6-sulfonic acid) diammonium salt solution and 140 mM potassium persulfate solution in equal quantities. The mixture was allowed to react for 12 h at room temperature in dark conditions. The solution was then diluted to obtain an absorbance between 1.1 ± 0.02 units at 734 nm. Fresh ABTS radical cation solution were prepared for each assay. Extracts in concentrations in the range 1–500 μg/mL were mixed with the ABTS radical cation solution in a 96-well plate. The absorbance was then measured at 734 nm after 30, 60, and 90 min of incubation. A linear standard curve of Trolox was made using amounts of Trolox between 0 and 250 µg. Results are expressed as µg of Trolox equivalents (TEq) per g of sample (TEq/g) [97].

#### 3.6.3. FRAP

A Ferric Reducing Antioxidant Power (FRAP) assay was carried out according to Jones et al. [54] with some modifications. Briefly, the FRAP working solution was prepared by mixing 100 mL acetate buffer (250 mM, pH 3.6), 10 mL 2,4,6-tris(2-pyridyl)-s-triazine (TPTZ) solution (10 mM in 40 mM HCl) and 10 mL FeCl3 (20 mM). 50 µL extracts, in different concentrations (1–500 μg/mL), were mixed with 200 µL of FRAP solution in a 96-well plate and incubated for 10 min at 37 °C. Then the absorbance was measured after 10, 20, 30, and 40 min at 593 nm. The linear standard curve of Trolox was prepared using Trolox amounts ranging from 0 to 250 µg. Results are expressed as µg TEq/g sample.

#### 3.6.4. Electrochemical Characterization of *N. alba* Extracts

The electrochemical investigations using cyclic voltammetry (CV) were employed for the characterization of *N. alba* leaf and root extracts. The electrochemical system consisted of an electrochemical cell (20 mL) with three electrodes: glass carbon electrode as working electrode, Ag/AgClsat (E = 0.194 V/NHE) as reference electrode and Pt wire as counter electrode. The measurements at room temperature were conducted with Bio-logic potentiostat/galvanostat SP-150 (Claix, France). The applied potential was E = ± 1 V vs. Ag/AgClsat, with different sweep rate potential between 10 to 1000 mV s^−1^. The working electrode was polished with BASi^®^ polishing kit (alumina and diamond slurries) followed by washing with methanol after each voltammetry experiment. The quercetin was also analyzed as a main component of the extracts. Fresh solutions of *N. alba* leaf and root extracts at 1 mg/mL and quercetin (10^−3^ M), were prepared in methanol. UV-Vis spectra were recorded before and after cyclic voltammetry experiment using SPECORD 210 PLUS double-beam spectrophotometer (Analytik Jena, Jena, Germany). Spectra were performed in the wavelength range of 200 to 700 nm using quartz cuvettes of 1 cm.

### 3.7. Statistical Analysis

All the experiments were performed in triplicate, independently, and the data presented were expressed as the mean ± standard deviation. Data were analyzed for statistical significance using the Microsoft Excel program, CompuSyn Software and GraphPad Prism Software.

## 4. Conclusions

Considering the imperative necessity of discovering new therapeutic agents, in this study, we evaluated for the *N. alba* aquatic plant the antifungal, antitumoral and antioxidant activities of methanolic leaf and root extracts.

The use of simple tests of seed germination allowed us to assess the toxic effects of *N. alba* extracts, and the toxicity studies exhibited no inhibitory effect on wheat seed germination. The confirmation of the non-toxic nature of the extracts was obtained from confocal laser scanning microscopy images.

The antioxidant activity evaluated by β-carotene bleaching, ABTS and FRAP assays was proven to be high, and the presence of the antioxidant flavonoid, quercetin, was confirmed by cyclic voltammetry.

The antifungal activity experiments showed that both extracts have high antifungal activity against the *C. glabrata* CBS 138 strain, acting as a fungistatic agent.

The *N. alba* leaf and root extracts showed a promising cytotoxic effect against ovarian A2780 and breast MCF7 cells and were less cytotoxic against noncancerous V79 cells indicating a more selective profile for cancer cells. In the cisplatin-resistant ovarian cells, *N. alba* leaf extract combined with cisplatin, inhibited the growth of the cells, suggesting a strong synergism in the growth inhibitory effect.

The results of the antifungal and antitumor activity of *N. alba* extracts, as well as studies on their impact on seed germination, suggest that the *N. alba* may act as an immune booster and prevent infection in immunosuppressed cancer patients. Further work is necessary to validate the benefits of *N. alba* extracts as human health promoters.

Altogether, the results presented here encourage us to continue our research into the exploitation of the bioactive potential of the *N. alba* species for the treatment of cancer and infection diseases.

## Figures and Tables

**Table 1:** Evaluation of different physiological parameters of wheat seed germination on treatment with *N. alba* extracts. Results are expressed as mean values ± standard deviation.

Physiological Parameters *	Concentration of the *N. alba* Extracts (µg/mL)								Control
	Leaf				Root				
	10	100	500	1000	10	100	500	1000	
**G %**	94.2 ± 0.24	94.6 ± 0.40	95.9 ± 0.91	97.4 ± 0.50	94.7 ± 0.52	96 ± 0.37	96 ± 0.15	97.4 ± 1.00	100 ± 0.00
**RRG %**	84.60 ± 1.20	101.13 ± 0.87	103.43 ± 1.13	103.55 ± 0.97	69.09 ± 0.73	91.76 ± 1.00	94.55 ± 0.76	100.07 ± 0.45	100 ± 0.00
**GI**	1.1 ± 0.10	0.9 ± 0.20	0.9 ± 0.25	0.9 ± 0.14	1.4 ± 0.10	1.0 ± 0.21	1.0 ± 0.50	1.0 ± 0.45	1.0 ± 0.00
**VI**	120.2 ± 0.43	146.2 ± 0.35	117.9 ± 0.10	113.8 ± 0.10	89.5 ± 0.24	137.6 ± 0.17	92.9 ± 0.24	123.7 ± 0.13	66.6 ± 0.14
**TI**	0.8 ± 0.12	0.6 ± 0.12	0.5 ± 0.24	0.6 ± 0.10	0.7 ± 0.43	0.5 ± 0.12	0.5 ± 0.11	0.8 ± 0.10	1.0 + 0.00

* G%—germination percentage; RRG%—relative root growth percentage; GI—germination index; VI—vigor index; TI—tolerance index.

**Table 2:** IC_50_ values of *N. alba* extracts obtained after 24 h incubation. Data were obtained from dose-response curves using GraphPad Prism ver. 5. The results are expressed in µg/mL.

*N. alba* Extracts	Tumor Cells *			
	V79	A2780	LNCaP	MCF-7
**Leaf**	367 ± 50	23.2 ± 3.0	274 ± 81	25.4 ± 5.9
**Root**	281 ± 59	19.4 ± 3.8	152 ± 47	18.1 ± 3.3

* V79—lung fibroblasts normal cells; A2780—ovarian tumor cells; LNCaP—prostate tumor cells; MCF-7—tumor breast cells.

**Table 3:** Interaction between *N. alba* extracts and cisplatin on the A2780 and A2780cisR cancer cell lines. Fa is the fraction affected by dose; CI is the combination index.

Drug (µg/mL)		A2780 *		A2780cisR *	
**Leaf**	**CisPt**	**Fa**	**CI**	**Fa**	**CI**
1000	100	0.03	4.85	0.12	1.20
500	50	0.01	1.03	0.10	0.44
200	20	0.04	1.22	0.06	0.08
100	10	0.02	0.35	0.07	0.05
10	1	0.70	2.07	0.60	0.57
**Root**	**CisPt**	**Fa**	**CI**	**Fa**	**CI**
1000	100	0.10	12.59	0.25	2.81
500	50	0.12	7.64	0.32	2.28
200	20	0.09	2.25	0.17	0.30
100	10	0.12	1.52	0.26	0.30
10	1	0.69	2.25	0.89	10.03

* A2780—ovarian tumor cells; A2780cisR—ovarian tumor cisplatin-resistant cells. Cisplatin = CisPt

**Table 4:** Antioxidant activity of *N. alba* extracts determined by BCB, ABTS and FRAP assays.

Assay *	*N. alba* Extracts					
	Leaf			Root		
	Inhibition Percent (%)	IC_50_ (µg/mL)	µg TEq/1 g	Inhibition Percent (%)	IC_50_ (µg/mL)	µg TEq/1 g
**BCB**	78.6 ± 0.19	33 ± 0.86	28,933 ± 0.89	90.1 ± 0.90	21 ± 0.55	79,371 ± 1.03
**ABTS**	77.0 ± 0.73	12 ± 0.11	1309 ± 0.33	78.2 ± 0.12	10 ± 0.10	1950 ± 0.41
**FRAP**	74.6 ± 0.13	30 ± 0.25	2245 ± 0.67	79.7 ± 0.13	13 ± 0.15	2407 ± 0.98

* BCB—β-Carotene bleaching; ABTS—2,20-azinobis (3-ethylbenothiazoline-6-sulfonic acid) diammonium salt solution radical cation; FRAP—Ferric Reducing Antioxidant Power.

**Table 5:** Identified compounds from *N. alba* leaf and root extracts and their biological properties.

No.	Identified Compounds from *N. alba* Extracts	Biologic Properties	References
**1**	HHDP-hexoside	antioxidant, anti-inflammatory, antitumor, and apoptotic properties; antibacterial activity against *E. coli, S. aureus, A. baumannii, P. aeruginosa*;	[59,60,61,62]
**2**	Quinic acid	induces cancer cell death by modulating the expression of Akt, phospho-Akt, and cell cycle pathway; anti-prostate cancer, attenuates Alzheimer’s disease	[63,64,65]
**3**	Vanillic acid	antibacterial, antioxidant and antihypertensive activities, α-glucosidase and tyrosinase inhibitory, effects against dextran sulfate sodium (DSS)-induced ulcerative colitis	[66,67,68,69]
**4**	Gallic acid	antiviral and antioxidant activities, anticancer activity by inducing apoptosis, downregulating genes involved in cell cycle and angiogenesis, and stimulating a cellular immune response	[24,70,71,72,73]
**5**	Castalin	antimicrobial, anti-inflammatory	[74]
**6**	Chlorogenic acid	inhibiting gene β-catenin and inducting genes GSK-3β, antispasmodic and antioxidant activities, inhibition of the HIV-1 integrase and inhibition of the mutagenicity of carcinogenic compounds	[75,76]
**7**	Corilagin	anticancer, anti-hyperalgesic, antioxidant, anti-inflammatory, hepatoprotective, and antitumor actions, induced apoptosis and autophagic cell death	[24,73,77,78]
**8**	Brevifolin	antioxidant, anti-inflammatory and anticancer	[61]
**9**	Caffeic acid	inhibiting gene β-catenin and inducting genes GSK-3β, antispasmodic and antioxidant activities	[75,76]
**10**	p-Coumaric acid	anti-inflammatory, anti-tyrosinase and antimicrobial activities, antispasmodic and antioxidant activities	[61,76,79]
**11**	Tannic acid	anticancer activity by inducing apoptosis, downregulating genes involved in cell cycle and angiogenesis, and stimulating a cellular immune response, Antioxidant and α-amylase inhibitory activities	[24,80,81]
**12**	Rutin	antioxidant activity, apoptosis, down regulating genes involved in cell cycle and angiogenesis antimetastatic, induces glutathione and glutathione peroxidase activities	[23,82,83]
**13**	Ellagic acid	anticancer activity by inducing apoptosis, downregulating genes involved in cell cycle and angiogenesis, and stimulating a cellular immune response; antioxidant and antiviral activities, inhibit α-glucosidase and α-amylase	[24,72,84,85]
**14**	Ellagic acid rhamnosyl	anticancer activity by inducing apoptosis, downregulating genes involved in cell cycle and angiogenesis, and stimulating a cellular immune response	[24,73]
**15**	Quercetin	apoptosis, down regulating genes involved in cell cycle and angiogenesis inhibited melanoma cell inhibits development of *Candida* spp.; antifungal	[23,39,59]
**16**	Ellagic acid-pentoside	anticancer activity by inducing apoptosis, downregulating genes involved in cell cycle and angiogenesis, and stimulating a cellular immune response, antioxidant activity	[24,73,84]
**17**	Naringenin	anticancer effect arrests cell development at the G0/G1 phase; inhibits *Candida* spp. growth	[39,43,60]
**18**	Naringin	anticancer effect arrests cell development at the G0/G1 phase; inhibits *Candida* spp. growth	[39,43,60]
**20**	Luteolin	anticancer associated with the induction of apoptosis, and inhibition of cell proliferation, metastasis and angiogenesis	[60,86]
**21**	Ferulic acid	synergistic effects against *Candida albicans*; anti-inflammatory and antioxidant activities, antispasmodic and antioxidant activities	[76,87]
**22**	Cinnamic acid derivative	anti-inflammatory, anti-tyrosinase and antimicrobial activities	[79]
**23**	Catechin	anticancer activity	[60,88]
**24**	Epicatechin	anticancer activity	[60,88]
**25**	Apigenin	anticancer effect by arresting cell development at the G0/G1 phase; the inhibition of phosphorylation of mitogen-activated protein kinase (MAPK)	[60]
**26**	Orientin	antioxidant, antiviral and antibacterial activities, anti-inflammatory neuroprotective or antidepressant-like effects	[89]
